# High ERα36 Expression Level and Membrane Location Predict Poor Prognosis in Renal Cell Carcinoma

**DOI:** 10.1097/MD.0000000000001048

**Published:** 2015-07-02

**Authors:** Qiang Wang, Wei Zhang, Jing Yang, Yu-Lin Liu, Ze-Xuan Yan, Zheng-Jun Guo, Yu-Jun Li, Xiu-Wu Bian

**Affiliations:** From the Institute of Pathology and Southwest Cancer Center (QW, JY, Z-XY, Z-JG, X-WB), Southwest Hospital, Third Military Medical University, Chongqing; Department of Pathology (QW, WZ); Department of Clinical Laboratory (Y-LL), The 401st People's Liberation Army Hospital; and Department of Pathology (Y-JL), Affiliated Hospital of Medical College, Qingdao University, Qingdao, China.

## Abstract

Estrogen receptor alpha 36 (ERα36), a truncated variant of ERα, is located in cytoplasm and membrane that is different from other nuclear receptors of ERα family. ERα36 is involved in progression and treatment resistance of a variety of carcinomas. However, the clinical and prognostic significance of ERα36 in renal tumors have not been fully elucidated.

Here, renal tumor tissues from 125 patients were collected and immunohistochemical stained with ERα36 antibody. ERα36 expression level and location in these cases were analyzed for their correlations with clinical characteristics. The differential diagnosis value was also assessed for benign and malignant renal tumors, as well as its prognostic value.

The results showed that membrane ERα36 expression was rarely detected in benign tumors but predominantly observed in malignant renal tumors. Kaplan–Meier analysis indicated that significant correlations of high ERα36 level and ERα36 membrane expression were correlated with both poor disease-free survival and overall survival. Univariate and multivariate analysis confirmed that both ERα36 high expression and membrane location can serve as unfavorable prognostic indicators for renal cell carcinoma.

It is thus concluded that membrane ERα36 expression is valuable for differential diagnosis of malignant renal tumors from benign ones. Both ERα36 high expression and membrane location indicate poor prognosis in renal cell carcinoma.

## Abstract 2

Supplemental Digital Content is available in the text

## INTRODUCTION

Most primary renal tumors are malignant, but it is difficult for a differential diagnosis of benign renal tumors from malignant ones, because of the complicated histological characters in renal tumors.^1,2^ Renal cell carcinoma (RCC) is the leading lethal urologic malignancy, which accounts for about 3% of malignant neoplasm.^3^ The common therapy for RCC is surgery, followed by chemotherapy or radiotherapy.^4^ However, high recurrence rate (20%–40%) is observed during these treatments.^5^ Local recurrence or distant metastasis usually leads to incurable disease of localized RCC. The lack of biomarkers for prognosis estimation may lead to poor clinical response.^4,6^ Hence, it is required to investigate the predictive biomarkers for differential diagnosis and targeting therapies for renal tumors.

Emerging proofs indicate that estrogens and their receptors play critical roles in various cancers and it is speculated that human kidney maybe also affected.^7^ The animal models of renal cancer that were established with estrogens exposure also confirmed that hormone/estrogen receptor (ER) complex participated in renal cell carcinoma initiation and progression.^8–10^ Two types of ERs, ERα and ERβ were investigated in clinical cases in previous studies.^11–14^ However, immunohistochemistry (IHC) study of tissue microarray (TMA) showed that ERα immunoreactivity was less than 10% of tumor cell nuclei.^15,16^ Another study found that estrogen-activated ERβ acted as a tumor suppressor in renal cell carcinoma.^12^ However, gene expression analysis of ER targeted genes in renal cell carcinoma demonstrated that ER signaling was closely associated with tumor progression.^17,18^ Therefore, hormone/ER signaling-related cancer progression is probably mediated by another ER variant.

ERα36 is a truncated variant of ERα, which was reported located in membrane and cytoplasm, rather than nuclei.^19^ It is participated in non-genomic estrogen signaling to promote cell proliferation.^20,21^ The expression of ERα36 is correlated poor prognosis in many kinds of carcinoma.^22–24^ In this study, we assessed the expression of ERα36 by IHC in renal tumors, and its association with clinicopathologic characteristics as well as clinical outcome. We further evaluated its differentiation and prognostic significance in renal tumors.

## METHODS AND MATERIALS

### Patients and Tumor Tissues

The retrospective study cohort consisted of 125 patients with primary renal tumors, who underwent surgical resection in the Affiliated Hospital of Qingdao University Medical College, and 401st Hospital, Shandong, China, between 2001 and 2013. Informed consent was obtained from each patient according to the research proposals approved by the local ethics committee of Qingdao University and 401st Hospital. Eligibility criteria included written informed consent and availability of tumor tissue, and follow-up data. For each patient, the following clinicopathologic information was collected, including age, sex, tumor size, TNM stage, presence of histological tumor necrosis, and Fuhrman grade. Clinical information was obtained by reviewing the medical records, by telephone or written correspondence, and by reviewing the death certificate. Follow-up information was updated every 6 months by telephone interview or questionnaire letters and was last done in January 2015.

### TMA and IHC

The IHC study was performed as previously described.^24^ ERα36 expression levels in 5 renal tumor tissues were studied by immunoblotting and qRT-PCR assays,^19^ which confirmed the IHC staining specificity (Supplemental Figure 1, http://links.lww.com/MD/A310). TMA was created from the formalin-fixed, paraffin-embedded tissue blocks of the patients. All samples were reviewed histologically by hematoxylin and eosin (HE) staining, and representative areas were marked on the paraffin blocks away from necrotic and hemorrhagic materials. Sections from the TMA blocks were cut at 4 μm. Primary antibody against human ERα36 (Shinogen, China) was applied for immunohistochemistry analysis. Antigen retrieval was performed in citrate buffer pH 6.0, then the sections were incubated overnight at 4°C with the primary antibody at 1:200. Next, they were rinsed with phosphate buffer solution (PBS) and incubated with the horseradish peroxidase-conjugated secondary antibody, followed by a rinse in PBS, incubation with diaminobenzidine staining, and counterstaining with hematoxylin blue. The negative control sections were incubated with control IgG in equal concentrations to the primary antibody, and known positive human breast cancer tissue was performed as positive control.

### Evaluation of ERα36 Immunohistochemical Staining

Representative IHC images in renal cell carcinoma tissues were collected at 40× objective with BX51 microscope (Olympus, Japan) and DP72 Camera (Olympus, Japan). The IHC staining level was assessed with German semiquantitative scoring system.^25^ The score for each sample was multiplied the staining intensity (0, no staining; 1, weak; 2, moderate; and 3, strong) and the percentage of tumor cells (0, 0%; 1, 1%–24%; 2, 25%–49%; 3, 50%–74%; 4, 75%–100%) at each intensity level, ranging from 0 (the minimum score) to 12 (the maximum score). The membrane/cytoplasm positive staining was determined by the subcellular location of the ERα36 positive granules. Generally, ERα36 positive granules, which arranged as cellular outlines, were diagnosed as membrane positive, whereas those with brown intracytoplasmic granules were diagnosed as cytoplasm positive. The IHC results were evaluated by 2 pathologists without the knowledge of patient outcome.

### Statistical Analysis

All data were analyzed using SPSS 19.0 software. The categorization was analyzed with the receiver-operating characteristic curve (ROC).^26^ The correlation of ERα36 and other potential clinical variables were assessed using Fisher exact test.^27,28^ Kaplan–Meier analysis with log-rank test was applied to compare survival curves.^29^ A univariate/ multivariate analysis was done using Cox proportional hazards model. Hazard ratios and their corresponding 95% confidence intervals were computed to provide quantitative information about the relevance of results of statistical analysis.^30^ All statistical tests were 2 sided and differences with a *P* value of 0.05 or less were considered to be statistically significant.

## RESULTS

### Patient Characteristics and Associations with ERα36 Expression

**FIGURE 1:**
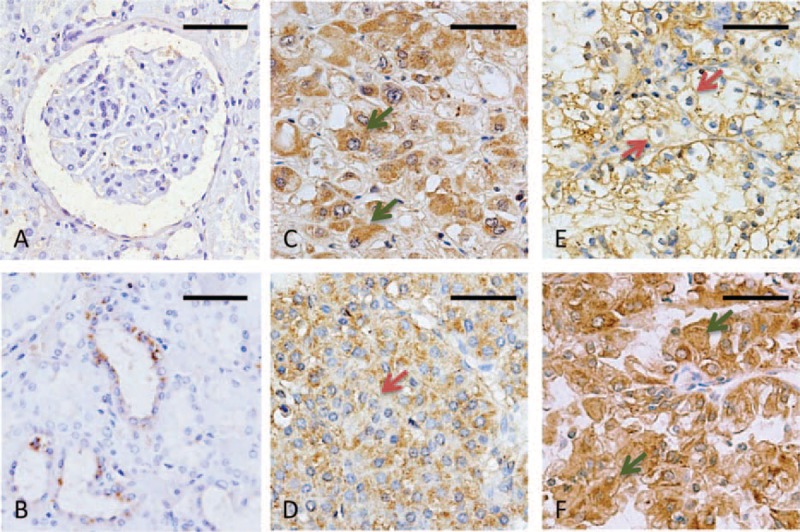
ERα36 expression in renal tumors (immunohistochemistry). (A, B) Low immunoreactivity was observed in the pericarcinous renal tissues: nephron (A) and renal tubules (B). (C, D) Most benign renal tumors showed dominant cytoplasm ERα36 expression (C). Only 1 case showed weak membrane location (D). (E, F) ERα36 positive staining was observed in the membrane (E) or cytoplasm (F) of renal cell carcinomas. Representative tumor cells positive for cytoplasm or membrane were shown with arrows (green arrows, cytoplasm; red arrows, membrane). Scale bar = 50 μm. ERα36 = estrogen receptor alpha 36.

A total of 99 patients with renal cell carcinoma were analyzed for ERα36 expression, as well as another 26 cases of diagnosed benign renal tumor. Immunohistochemical staining showed that the pericarcinous renal tissues were observed with low ERα36 immunoreactivity. ERα36 expression was rarely observed in nephron (Figure 1A), but found in some renal tubules (Figure 1B). However, ERα36 expression was found in benign renal tumors (Figure 1C, D). High ERα36 expression was also observed in primary renal cell carcinoma, which was predominantly located in the cytoplasm and membrane of cancer cells (Figure 1E, F). In the cancer cell bulks, ERα36 expression was distributed primarily in a hierarchical pattern (Figure 1F).

### Comparison of ERα36 Expression in Benign and Malignant Renal Tumors

**FIGURE 2:**
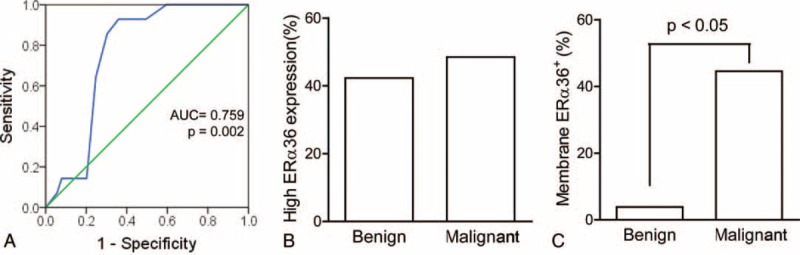
Comparison of ERα36 expression in benign and malignant renal tumors. (A) A receiver-operating characteristic curve was analyzed for a reasonable cutoff point, which support the cutoff point, was score = 4.5 (low: score ≤4; high: score ≥5). The area under the curve (AUC) was 0.759 (*P* = 0.002). (B) Percentage of ERα36^high^ in benign and malignant renal tumors. (C) Percentage of membrane ERα36 expression in benign and malignant renal tumors. Data were analyzed with χ^2^ test. ERα36 = estrogen receptor alpha 36.

To determine the differential diagnosis value of ERα36 in renal tumors, a comparison was performed between renal cell carcinoma and benign tumors. The primary tumors were categorized into 2 groups according to the IHC scores: high (score ≥5); low (score ≤4) (Figure 2A). No significant difference in the percentage of ERα36^high^ cases was observed between malignant and benign tumors (48.5% vs 42.3%, Figure 2B). Of interest, a remarkable difference was observed in ERα36 location between benign and malignant tumors. Membrane location of ERα36 was rarely observed in benign tumors rather than malignant ones (3.5% vs 46.5%, Figure 2C). ERα36 expression in benign tumors was characteristically located in the cytoplasm (Figure 1C), only 1 benign tumor showed weak membrane positive staining (Figure 1D), whereas higher percentage of membrane positive was observed in malignant ones (Figure 1E). Thus, ERα36 expression location may be served as a differential diagnosis marker for renal tumors.

### Relationship Between ERα36 Expression and Clinical Features

The relationships between ERα36 expression levels and clinical features in renal cell carcinoma were listed in Table 1. Totally 48 cases were observed with high ERα36 expression. ERα36 expression level was statistically associated with tumor size (*P* = 0.022), clinical stage (*P* = 0.029), and necrosis (*P* = 0.018). ERα36 high expression was correlated with larger tumor size, late clinical stage and more necrosis in tumor tissue. However, we failed to detect significant correlations between ERα36 expression level and other clinical characteristics, including age, sex, resection procedure, histological subtype, and Fuhrman grade.

**TABLE 1:**
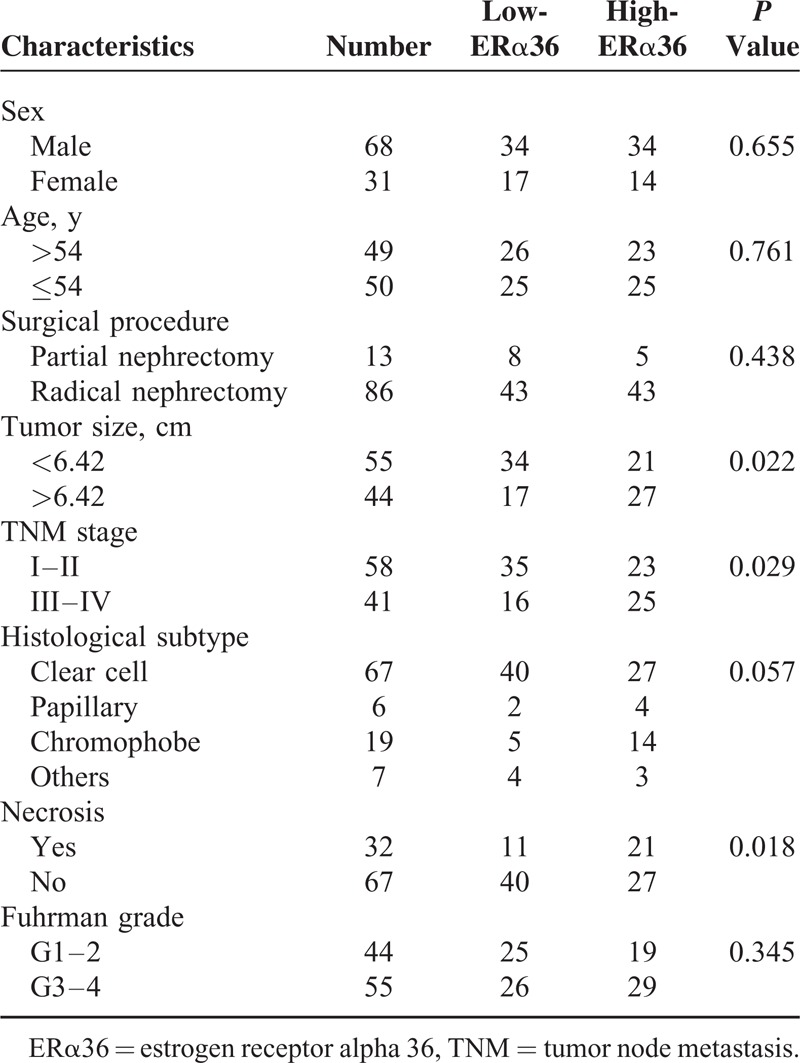
Correlations of ERα36 Expression Level and Clinical Characteristics of Renal Cell Carcinoma

Furthermore, the relationships between ERα36 location and clinical features were shown in Table 2. Dominant membrane ERα36 expression was found in 41 cases, and cytoplasm expression in 51 cases (7 cases which scored 0 were excluded). Different location of ERα36 was only correlated with necrosis (*P* = 0.002). More necrosis was observed in membrane ERα36 expression cases. No significant correlation was found between ERα36 location and other clinical characteristics. Moreover, no significant correlation was observed between ERα36 expression level or subcellular location and ERα66 expression (Supplemental Figure 2, http://links.lww.com/MD/A310, and Supplemental Table 1, http://links.lww.com/MD/A310).

**TABLE 2:**
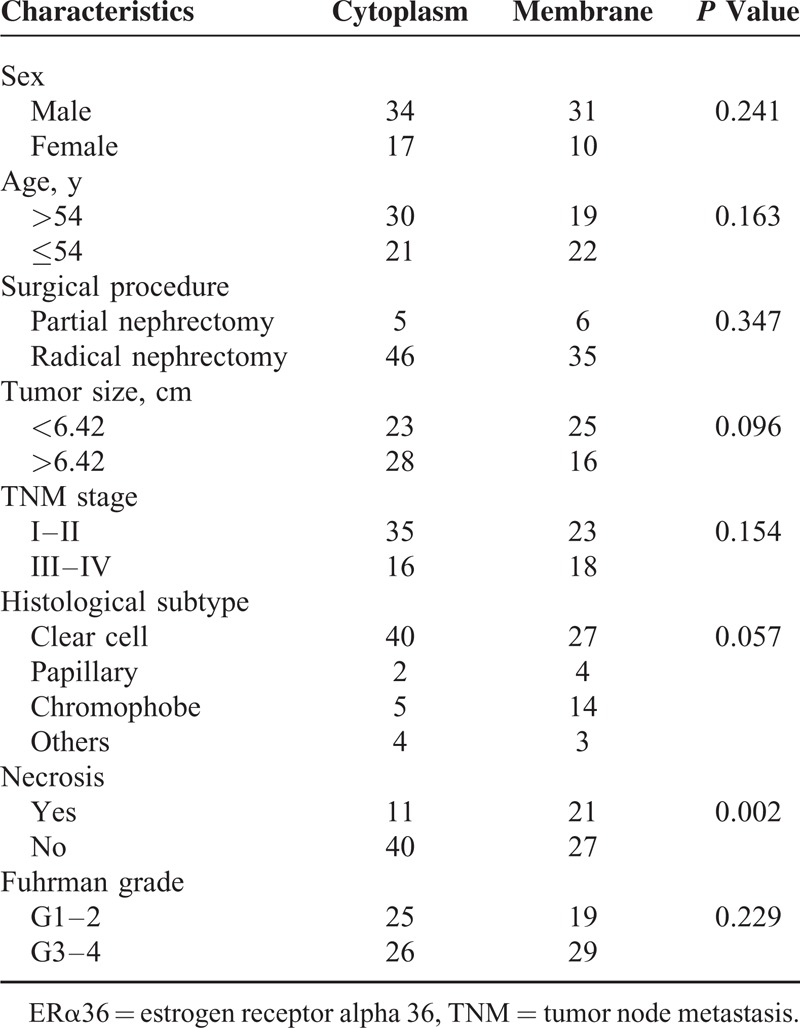
Correlations of ERα36 Location and Clinical Characteristics

### ERα36 Expression Correlated With Poor Clinical Outcome

**FIGURE 3:**
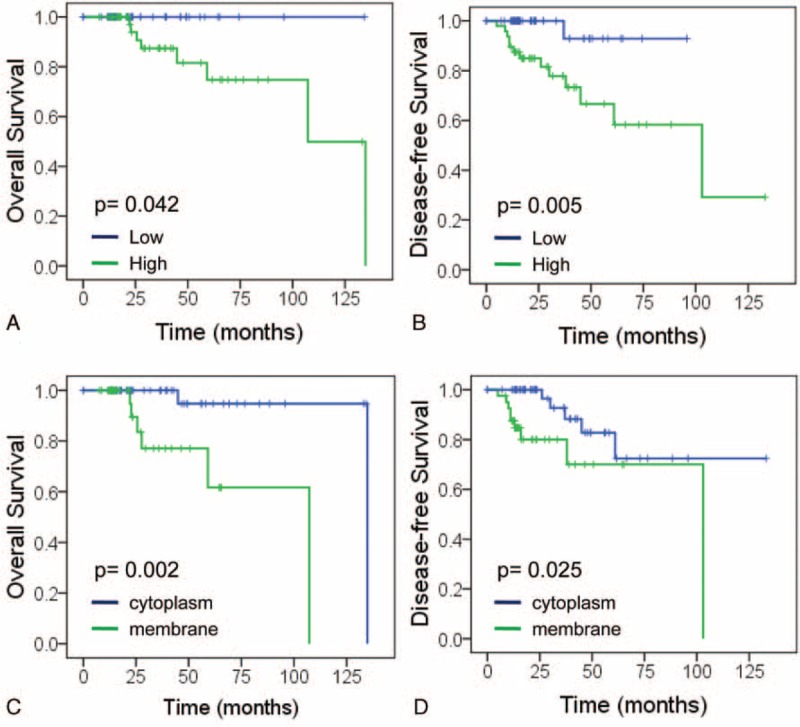
Effect of ERα36 expression on patient prognosis. (A, B) High ERα36 expression is associated with poor prognosis of patients: overall survival (A) and disease-free survival (B). (C, D) Membrane ERα36 expression is associated with poor prognosis of patients: overall survival (C) and disease-free survival (D). ERα36 = estrogen receptor alpha 36.

Follow-up information was available for all patients and the median period was 40.9 months (range: 21–135 months). During the follow-up period, carcinoma progression was found in 14 patients (14.1%). Kaplan–Meier curves were analyzed to show that ERα36 high expression was statistically correlated with both poor overall survival (OS, *P* = 0.042) and disease-free survival (DFS, *P* = 0.005) in renal cell carcinoma (Figure 3A, B). More importantly, worse prognosis was also observed in the patients with ERα36 membrane expression than those predominately in cytoplasm in both OS (*P* = 0.002) and DFS (*P* = 0.025) (Figure 3C, D).

### Prognostic Significance of ERα36 Expression

Cox univariable and multivariable proportional hazard models were constructed to evaluate the independent prognostic significance of ERα36 expression levels and locations with clinical characteristics including age, sex, tumor size, clinical stage, tumor necrosis, and Fuhrman grade. The results of Cox univariate analysis showed that ERα36 high expression was a significant predictor for shorter DFS in renal cell carcinoma, independent of other factors (*P* = 0.017, Table 3). Moreover, the membrane ERα36 expression was also a significant predictor for both shorter DFS and OS (*P* = 0.040, *P* = 0.020, Table 4).

**TABLE 3:**
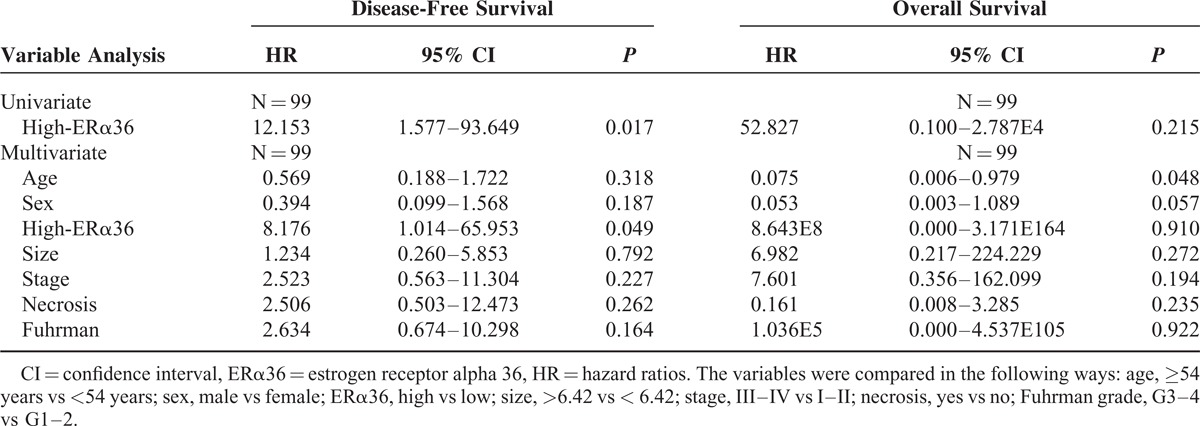
Univariate and Multivariate Analyses of Disease-Free Survival and Overall Survival (ERα36 Expression Level)

**TABLE 4:**
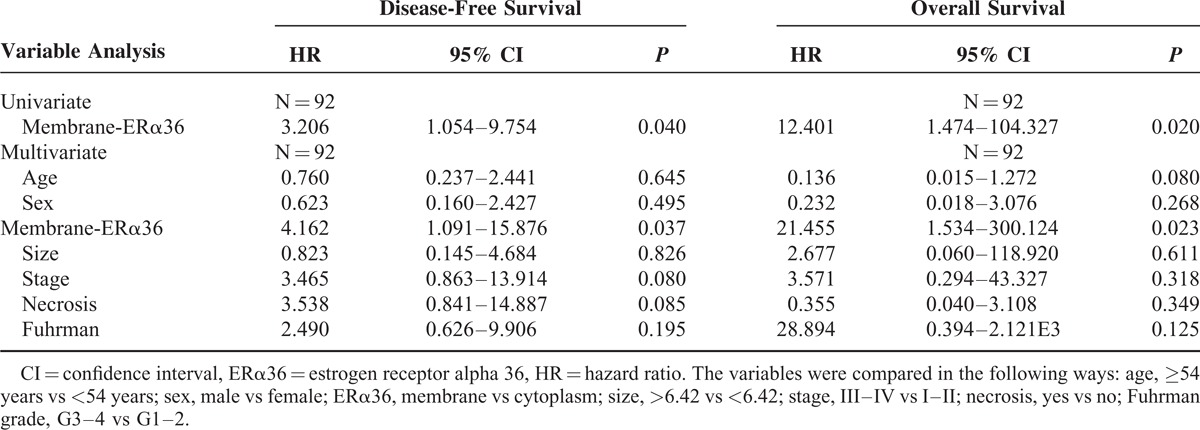
Univariate and Multivariate Analyses of Disease-Free Survival and Overall Survival (ERα36 Membrane Location)

Multivariate Cox regression analysis showed that ERα36 high expression was significantly correlated with worse DFS (*P* = 0.049, Table 3), but not correlated with OS (*P* = 0.910, Table 3). More importantly, significant worse DFS and OS were observed in the patients with ERα36 membrane positive patients relative to the cytoplasm positive ones (*P* = 0.037, *P* = 0.023, Table 4).

## DISCUSSION

Dysregulated estrogen signaling contributes to the initiation and progression of renal cell carcinomas,^21,31^ but the mechanism has not been well established.^32,33^ Our study here investigated the expression of ERα36 in renal tumors, which provide further insight in this field. ER expression is observed in both reproductive and nonreproductive tissues and cancer tissues.^34^ We provided evidences that ERα36 expression was correlated with poor prognosis in renal cell carcinoma, which indicated ERα36 may be involved in tissue responsiveness to estrogens for carcinogenesis and progression.

High expression of ERα36 was an independent predictor for poor prognosis in renal cell carcinoma. Different from the 66KDa ERα (ERα66), high ERα36 expression was observed on the plasma membrane and cytoplasm of renal cancer specimens.^24,35^ As a truncated isoform of ERα66, ERα36 gene completely matches with exon2 to exon6 of ERα66 gene.^19,36^ Some epitopes are shared by ERα36 and ERα66 proteins, which explain the cytoplasm pattern of ERα66 expression that was observed in renal carcinoma tissues.^15^ Here, the specific antibody for ERα36 was generated from the unique peptide in ERα36-C terminal. Molecular tests further guaranteed the specificity in IHC study in the tumor tissues. High levels of ERα36 expression were significantly correlated with necrosis in renal cell carcinoma, which is one of the most important prognostic factors. Further analyses were also confirmed that high ERα36 expression was correlated with increased metastasis and poor prognosis. Therefore ERα36 expression can be used as an independent predictive marker for the progression of renal cell carcinoma.

More importantly, membrane ERα36 expression is correlated worse prognosis relative to cytoplasm positive, which indicated that non-genomic estrogen signaling mediated by ERα36 may be involved in renal cell carcinoma progression. Different from those traditional nuclear receptor variants, ERα36 is located on membrane and cytoplasm as reported in previous studies.^37,38^ The plasma membrane-localized ERα36 was proposed to transduce membrane-initiated estrogen signaling.^39^ When estradiol binds to the cell surface receptor, a rapid generation of cAMP is stimulated. The non-genomic estrogen signaling is transduced to activate RNA and protein synthesis,^34^ which regulates various physiopathological processes for carcinogenesis and progression,^31,40^ such as promoting cell proliferation and invasion.^41^ Thus, membrane located ERα36 and related signaling maybe responsible for tumor progression of renal cell carcinoma. However, further studies for the mechanism are required in the future.

Accurate classification is crucial for both diagnosis and therapeutic intervention in renal tumors. However, majority of renal tumors have unusual morphology that renders classification challenging,^42^ such as the differential diagnosis of renal tumors with tubulopapillary features includes metanephric adenoma and papillary renal cell carcinoma.^1,2^ Accurate classification relies on careful examination of clinical and pathological features and immunohistochemical characteristics. Here, we evaluated ERα36 subcellular location for renal tumor classification and found that ERα36 membrane location was rarely observed in benign tumors, which provide useful criteria for accurate diagnosis differentiation in renal tumors.

Different ERα variants play important roles for estrogen signaling dysregulation. No significant correlation was observed between ERα36 and ERα66 in our study. However, other ERα variants (such as ERα46) were not included in our IHC study because of the limitation of specific antibody for them. Further study is still needed for the interaction between different variants. Taken together, membrane located ERα36 may act a critical role for renal cell carcinoma initiation and progression. IHC staining for ERα36 can provide valuable information for diagnosis, prognostication, and personalized treatment of renal tumors.
